# Evaluating lifetime nitrogen use efficiency of dairy cattle: A modelling approach

**DOI:** 10.1371/journal.pone.0201638

**Published:** 2018-08-02

**Authors:** Andreas Foskolos, Jon M. Moorby

**Affiliations:** Institute of Biological, Environmental and Rural Sciences, Gogerddan, Aberystwyth, United Kingdom; University of Illinois, UNITED STATES

## Abstract

The increased nitrogen (N) use efficiency in cattle farming is proposed as a key action to improve N management and reduce the environmental impact of cattle farming systems. Most attention has been given to lactating cow nutrition, excluding the elements of fertility, disease, and the non-lactating animals within the herd. Therefore, the aim of the current study was to develop a herd-level simulation model incorporating these elements to assess dairy farm N use efficiency. We developed a cattle N use efficiency (CNE) model with six primary compartments: (i) heifer growth, (ii) heifer removal, (iii) pregnancy, (iv) cow removal, (v) disease and fertility, and (vi) milk production. The CNE model calculates N loss or gain for each compartment, and then calculates the lifetime N loss or gain taking into account the replacement rate (*rep*) and/or the corresponding number of lactations in a herd (Lact = 1/*rep*). Finally, three N use efficiencies were estimated: (i) ReplNE: replacement cattle N use efficiency, (ii) LactNE: lifetime N use efficiency for lactation, and (iii) LNE: lifetime N use efficiency. The sensitivity of the model to variation in farm- and animal-related input values was evaluated using Monte Carlo simulation. Values for a model dairy farm were used based on published data reflecting typical dairy farming practices in the United Kingdom. To assist reporting net values of main N outputs, a dairy herd of 100 lactating cows was modelled. Productive N outputs (1000s of kg) over the course of an animal’s lifetime, partitioned into milk and meat, were dominated by milk production (89% of total N output). We estimated a mean ReplNE of 23.7%, affected most by the last stage of heifer growth. The Monte Carlo sensitivity analysis suggested that variation in time to first calving (T_1stCal_) might cause larger changes on ReplNE than variation in feed. The sensitivity analysis revealed a strong positive correlation between dietary oriented milk N use efficiency (MNE) and LactNE and LNE (*r* = 0.99 and 0.97 for LactNE and LNE, respectively). However, our study highlighted two other model variables that affected LNE. Variation in calving interval (CI; *r* = −0.15) and T_1stCal_ (*r* = −0.15) may cause measurable reductions of overall LNE. The first is an indicator of lactating cattle fertility, and the second an indicator of replacement cattle growth and fertility efficiency. In conclusion, with the current study we provided a dairy cattle herd model that is sensitive in elements of diet, fertility and health. Lifetime N use efficiency of dairy cattle is dominated by MNE, but we detected specific non-diet related variables that affect ReplNE, LactNE and LNE.

## Introduction

Increasing public awareness about environmental issues and the environmental impacts of dairy production challenge the dairy industry to perform in a more environmentally responsible way [1, 2]. Nitrogen (N) is an essential component of human food production, determining the productivity of crops and animals in fertilizers and feeds [3]. However, its extensive use has led to the so-called N cascade phenomenon, which refers to the circulation of anthropogenic N in ecosystems causing multiple effects on atmospheric, freshwater and marine environments [4–6]. European data suggest that agriculture is the main contributor to this phenomenon accounting for approximately 78% of total N entering the ecosystem [7] and an increase in the efficiency of N use in crop and animal production is proposed as a key action to improve N management [8].

Nitrogen use efficiency can be defined at the levels of the animal, the farm, and the entire agricultural supply chain. In milk production, milk N use efficiency (MNE), defined as the amount of milk N produced relative to N intake at an individual dairy cow level, is commonly used [9–11]. However, this approach focuses almost exclusively on the lactating cow and her nutrition, and generally excludes elements of fertility and disease as well as growing and non-lactating animals within the herd. Animal disease and fertility status affect milk production and it is possible to quantify these effects [12–15]. Moreover, growing animals can represent a significant proportion of animals in the production system [16]. Calves are a required consequence of milk production from cows, and contribute to herd replacements and to meat production. Therefore, besides the economic cost of heifer rearing, there is an impact on overall herd N use efficiency. Gross N use efficiency of milk-fed calves between 39 and 50%, and of growing heifers between 20 and 28% were reported [17]. Various studies have evaluated N use efficiency either at a cow level [9, 18] or at a farm level [15, 19, 20]. As a result, relative N use efficiency at a system level has been proposed [21]. However, none of the previous studies have incorporated elements of performance, fertility, and diseases of lactating and replacement cattle into a single model. Therefore, the aim of the current study was to develop a herd-level simulation model incorporating these elements to assess dairy cattle lifetime N use efficiency.

## Materials and methods

### Model description

**Fig 1:**
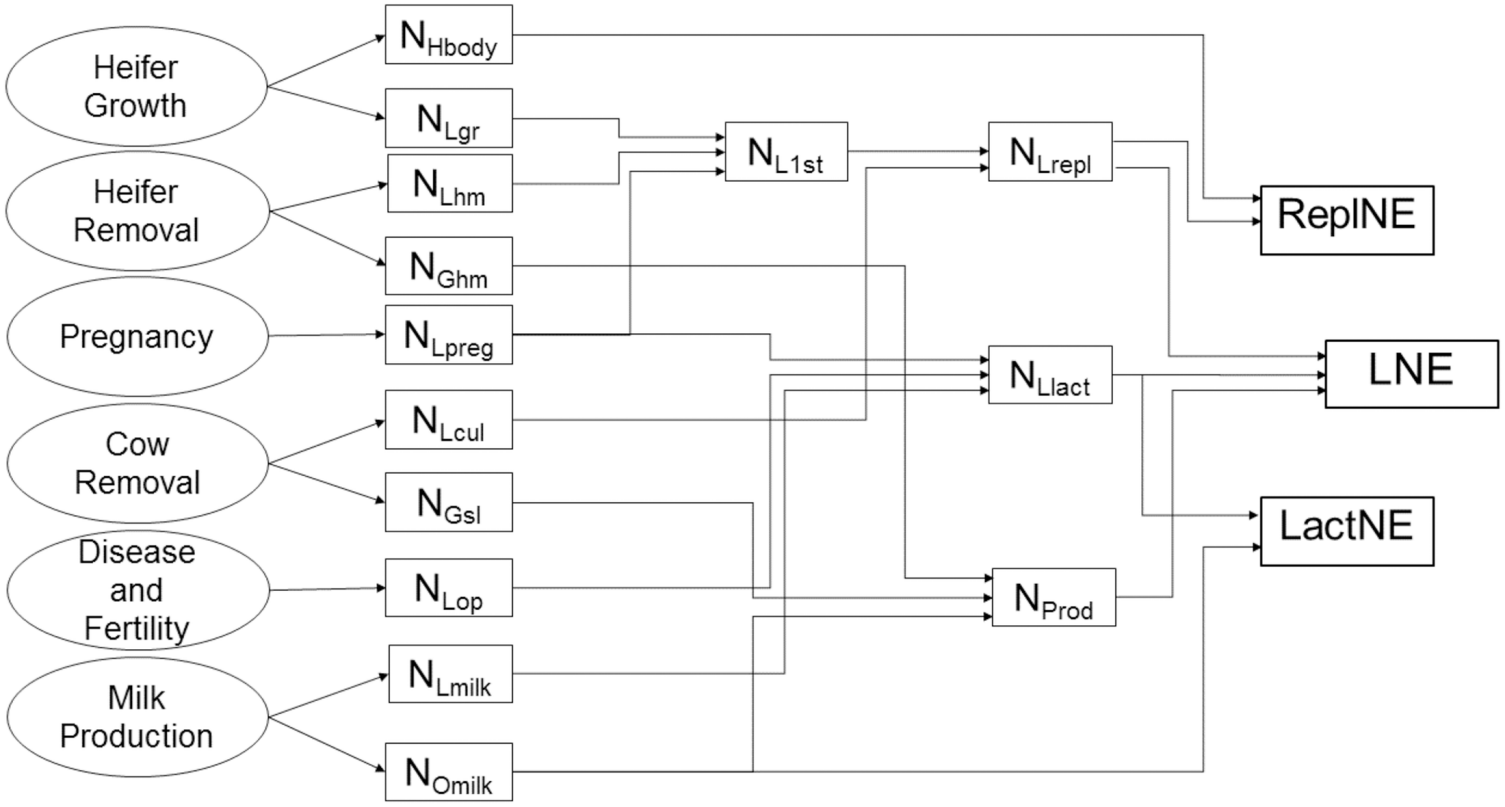
Schematic description of cattle nitrogen (N) use efficiency model (CNE) for dairy cattle. Where, N_Hbody_: N retained in heifer’s body; N_Lgr_: N lost for heifer growth; N_Lhm_: N lost due to heifer mortality; N_Ghm_: N gained from culled heifers; N_Lpreg_: N lost during pregnancy; N_Lcul_: N losses due to cattle culling; N_Gsl_: N gain due to sold cattle; N_Lop_: opportunity N losses; N_Omilk_: cumulative milk N output; N_Lmilk_: N lost due to milk production; N_L1st_: N lost from birth to first calving; N_Lrepl_: N lost for replacement cattle; N_Llact_: N losses in lactation for lifetime; N_Prod_: Produced N; ReplNE: replacement N use efficiency; LactNE: lactation N use efficiency; LNE: lifetime N use efficiency.

The dairy cattle N use efficiency (CNE) model was developed as a spreadsheet using Microsoft Excel and consists of six primary compartments: (i) heifer growth, (ii) heifer removal, (iii) pregnancy, (iv) cow removal, (v) disease and fertility, and (vi) milk production. Other potential farm parameters, such as the use and fate of N in manures, fertilisers, and male and surplus heifer calves sold off farm, were not considered. The definitions and abbreviations of model entities are presented in Table 1 and a schematic description of the CNE model is given in Fig 1.

**Table 1:** Definition of entities used in dairy cow nitrogen use efficiency (CNE) model.

Entity	Unit	Level	Definition
**Heifer growth and removal**			
BW_100_	kg	animal	Heifer at 100 kg of body weight
BW_1stCal_	kg	animal	Body weight at first calving
BW_1stSer_	kg	animal	Body weight at first service
BW_B_	kg	animal	Body weight at birth
BW_c_	kg	animal	Body weight of heifer at 6 months of age
BW_n_	kg	animal	Body weight of neonatal calf
CPreq	kg	animal	Crude protein requirements for heifer growth
m_c_	N/A^a^	herd	Calf mortality rate
m_h1stCal_	N/A	herd	Heifer mortality rate from first service to first calving
m_h1stSer_	N/A	herd	Heifer mortality rate from six months of age to first service
m_n_	N/A	herd	Neonatal mortality rate
m_p_	N/A	herd	Perinatal mortality rate
MP:CP_1stCal_	N/A	animal	Metabolizable protein to crude protein ratio at first calving
MP:CP_1stSer_	N/A	animal	Metabolizable protein to crude protein ratio at first service
MPGrowth	kg	animal	Metabolizable protein requirements for growth
N_6.25_			Protein to nitrogen conversion constant (N = protein/6.25)
N_FeedReq_	kg	animal	Total feed nitrogen required for heifer’s growth
N_FeedReq_BW100_	kg	animal	Feed nitrogen required for calf growth from weaning to reach 100 kg of body weight
N_FeedReq_c_	kg	animal	Feed nitrogen required for calf growth
N_FeedReq_h1stCal_	kg	animal	Feed nitrogen required for heifer growth from first service to first calving
N_FeedReq_h1stSer_	kg	animal	Feed nitrogen required for heifer growth from 100 kg of body weight to first service
N_FeedReq_n_	kg	animal	Feed nitrogen required for neonatal growth
N_FeedReq_w_	kg	animal	Feed nitrogen required for calf weaning
N_G1stCal_	kg	animal	Nitrogen gained from heifers removed between first service and first calving
N_G1stSer_	kg	animal	Nitrogen gained from heifers removed between six months of age and first service
N_Ghm_	kg	animal	Nitrogen gained from culled replacement heifers
N_Hbody_	kg	animal	Nitrogen retained in heifer’s body
N_Lgr_	kg	animal	Nitrogen lost for heifer growth
N_Lhm_	kg	animal	Nitrogen lost due to heifer mortality
N_Lm_	kg	animal	Total nitrogen losses due to heifer mortality
N_Lm1stCal_	kg	animal	Nitrogen lost due to heifer mortality to first calving
N_Lm1stSer_	kg	animal	Nitrogen lost due to heifer mortality to first service
N_Lmc_	kg	animal	Nitrogen lost due to calf mortality
N_Lmn_	kg	animal	Nitrogen lost due to neonatal mortality
N_Lmp_	kg	animal	Nitrogen lost due to perinatal mortality
P_%B_	%	animal	Cattle body protein content
s_h1stCal_	N/A	herd	Sold rate (proportion of animals sold for meat out of total animals removed) of heifers between first service and first calving
s_h1stSer_	N/A	herd	Sold rate of heifers between six months of age and first service
T_100_	day	animal	Age when body weight reaches 100 kg
T_1stCal_	day	animal	Age at first calving
T_1stSer_	day	animal	Age first service
T_c_	day	animal	Heifer at 6 months of age
T_n_	day	animal	Age of neonatal calf
T_w_	day	animal	Age at weaning
**Pregnancy**			
N_Calf_	kg	animal	Nitrogen in calf’s body
N_Lpreg_	kg	animal	Nitrogen lost during pregnancy
N_PregReq_	kg	animal	Feed nitrogen required for pregnancy
**Cow removal**			
BW_M_	kg	animal	Mature body weight
N_Gsl_	kg	animal	Nitrogen gain due to culled cows
N_Lcul_	kg	animal	Nitrogen losses due to cattle culling
s_c_	N/A	herd	Sold rate of dairy cows
**Disease and fertility**			
CI	day	animal	Calving interval
M_LCI_	Kg	animal	Milk lost due to extended calving interval
M_Lcm_m_	Kg/lactation	animal	Milk lost due to mild milk clinical mastitis
M_Lcm_s_	Kg/lactation	animal	Milk lost due to severe milk clinical mastitis
M_LD_	Kg/lactation	animal	Cumulative milk lost due to diseases
M_Ldl_	Kg/lactation	animal	Milk lost due to digital lameness
M_Lil_	Kg/lactation	animal	Milk lost due to interdigital lameness
M_Lmf_m_	Kg/lactation	animal	Milk lost due to mild milk fever
M_Lmf_s_	Kg/lactation	animal	Milk lost due to severe milk fever
M_Lop_	kg	animal	Milk lost due to disease and fertility problems
M_Lpm_	Kg/lactation	animal	Milk lost due to perinatal calf mortality
M_Lrp_	Kg/lactation	animal	Milk lost due to retained placenta
M_Lsu_	Kg/lactation	animal	Milk lost due to sole ulcer
M_Lvd_	Kg/lactation	animal	Milk lost due to vulval discharge
N_Lop_	kg	animal	Opportunity N losses
r_pm_	%	animal	Risk factor for perinatal calf mortality
r_cm_m_	%	animal	Risk factor for mild milk clinical mastitis
r_cm_s_	%	animal	Risk factor for severe milk clinical mastitis
r_dl_	%	animal	Risk factor for digital lameness
r_il_	%	animal	Risk factor for interdigital lameness
r_mf_m_	%	animal	Risk factor for mild milk fever
r_mf_s_	%	animal	Risk factor for severe milk fever
r_rp_	%	animal	Risk factor for retained placenta
r_su_	%	animal	Risk factor for sole ulcer
r_vd_	%	animal	Risk factor for vulval discharge
**Milk Production**			
MNE	g/g	herd	Milk nitrogen use efficiency
MY	kg	animal	Annual milk yield
N_6.38_			Milk protein to nitrogen conversion constant (N = milk protein/6.38)
N_Lmilk_	Kg/lactation	animal	Nitrogen lost due to milk production
N_Omilk_	Kg/lactation	animal	Cumulative milk nitrogen output
P_%_	%	herd	Milk protein content
**Herd Level**			
Lact		herd	Lactations (1 / cattle replacement rate)
*n*		herd	Lactating cattle in herd
N_L1st_	kg	herd	Nitrogen lost from birth to first calving
N_Llact_	kg	herd	Nitrogen losses in lactation for lifetime
N_LmilkLT_	kg	herd	Nitrogen lost for milk in lifetime
N_LopLT_	kg	herd	Opportunity nitrogen losses in lifetime
N_LpregLT_	kg	herd	Nitrogen lost for pregnancy in lifetime
N_Lrepl_	kg	herd	Nitrogen lost for replacement cattle
N_OmeatLT_	kg	herd	Nitrogen output in meat for lifetime
N_OmilkLT_	kg	herd	Nitrogen output in milk for lifetime
N_Prod_	kg	herd	Produced nitrogen
N_ReplBW_	kg	herd	Nitrogen retained in replacement cattle body
*rep*	N/A	herd	Cattle replacement rate
**Efficiency**			
LactNE	%	herd	Lactation nitrogen use efficiency
LNE	%	herd	Lifetime nitrogen use efficiency
ReplNE	%	herd	Replacement nitrogen use efficiency

^a^ N/A: not applied. This refers to proportions that have the same units in both parts of the ratio (e.g. cow/cow)

#### Heifer growth

For the heifer growth compartment, the equations of the 2001 Dairy NRC model were used [22] to calculate N loss or gain. Four stages of growth were considered: (i) from birth to weaning, (ii) from weaning to BW_100_, (iii) from BW_100_ to first service, and (iv) from first service to first calving. For the stages i and ii, N requirements (in the 2001 Dairy NRC model denoted as CPreq; currently expressed in N using the N_6.25_ factor) at birth, weaning and BW_100_ were calculated assuming daily BW gain of 400, 800 and 800 g, respectively, for calves fed milk replacer and starter diets, or weaned calves fed solid diets [22]. Cumulative amounts of N required for each stage (N_FeedReq_w_, N_FeedReq_BW100_) were calculated as the area under the interpolated line assuming N requirement was a linear function of time (T_W_, T_100;_ measured in days). Similarly, for the following two stages (iii and iv) MPGrowth (denoted as such by the NRC model) was calculated at BW_100_, first service, and first calving based on corresponding live weights (BW_100_, BW_1stSer_ and BW_1stCal_) and assuming net energy for growth from diets of 5.61, 9.63 and 12.98 MJ/d, respectively, to allow shrunk body weight gains higher than 0.6 kg/d. Cumulative MPGrowth was calculated as the area under the interpolated line assuming MPGrowth requirement was a linear function of time (T_1stSer_ and T_1stCal;_ measured in days). To convert from metabolizable protein (MP) requirements to crude protein (CP) inputs, MP:CP constants were used (MP:CP_1stSer_, and MP:CP_1stCal_, respectively). Metabolizable protein in ruminants comprises undegraded feed protein and microbial protein that leaves the rumen. Both undegraded feed and microbial protein are feedstuff- and animal-specific and vary depending on the animal’s diet and stage of growth. Mechanistic models, such as the 2001 Dairy NRC and the Cornell net carbohydrate and protein system (CNCPS) [18, 23] may be used to estimate CP intake and corresponding MP supply of a given diet and stage of growth; then, the ratio between these two will be the MP:CP constants. After converting to CP, N_FeedReq_h1stSer_ and N_FeedReq_h1stCal_ were calculated using N_6.25_. Finally, overall N_FeedReq_ was estimated as the sum of all stages:

$${\text{N}}_{\text{FeedReq}}={\text{N}}_{\text{FeedReq}\_\text{w}},+{\;\text{N}}_{\text{FeedReq}\_\text{BW}100}+{\text{N}}_{\text{FeedReq}\_\text{h}1\text{stSer}}+{\text{N}}_{\text{FeedReq}\_\text{h}1\text{stCal}}$$ (1)

Total N retained in a heifer’s body was calculated assuming 16% P_%B_ [24, 25] and N_6.25_:

$${\text{N}}_{\text{Hbody}}={\text{BW}}_{1\text{stCal}}\text{x}\left({\text{P}}_{\%\text{B}}/100\right)/{\text{N}}_{6.25}$$ (2)

Finally, N losses for growth were calculated with the following equation:

$${\text{N}}_{\text{Lgr}}={\text{N}}_{\text{FeedReq}}-{\text{N}}_{\text{Hbody}}$$ (3)

#### Heifer removal

Losses or gains of N due to calf and heifer removal from the herd were calculated for five lifetime stages. Causes of removal included mortality (death) and culling (voluntary removal due to either productive issues related to health problems, infertility, or any other negative reason, or sale as healthy animals). Animal removals at five different stages were considered: (i) perinatal: stillbirths and mortality within the first 24 h of birth of male and female calves; (ii) neonatal: the number of female calves that died or were euthanized between 24 h and 28 d of age; (iii) calf: the number of female calves that died or were euthanized between 1 and 6 months of age; (iv) heifers to first service: the number of heifers that died or were culled between 6 months old and the commencement of breeding (defined as the time of first insemination, first contact with a bull, or first embryo transfer); and (v) heifers to first calving: the number of heifers that died or were culled between the first breeding service and first calving. This last stage included those animals that failed to conceive. Losses of N to reach an animal a specific growth stage were calculated based on specific mortality rates (*m*_*n*_, *m*_*c*_, *m*_*h1stSer*_ and *m*_*h1stCal*_) and the N_FeedReq_ for each stage (N_FeedReq_n_, N_FeedReq_c_, N_FeedReq_h1stSer_ and N_FeedReq_h1stCal_) with the exception of N_Lmp_. These were calculated by the heifer growth sub-model using specified BW (BW_n_, BW_c_, BW_h1stSer_ and BW_h1stCal_) and times (T_n_, T_c_, T_h1stSer_ and T_h1stCal_) of dead or culled heifers in each stage. For the perinatal stage it was assumed that calves were not fed and N_Lmp_ was therefore based on *m*_*p*_ and BW_B._

To differentiate between system N losses and gains from heifers removed from the herd of the stages “heifers to first service” and “heifers to first calving” sold constants were used to represent the proportion of animals sold at market (*s*_*h1stSer*_ and *s*_*h1stCal*_; proportion of heifers sold for meat out of total heifers removed). The equations were:

$${\text{N}}_{\text{Lmp}}={\text{BW}}_{\text{B}}\times m_{p}\times \left({\text{P}}_{\%\text{B}}/100\right)/{\text{N}}_{6.25}$$ (4)

$${\text{N}}_{\text{Lmn}}=m_{n}\times {\text{N}}_{\text{FeedReq}\_\text{n}}$$ (5)

$${\text{N}}_{\text{Lmc}}=m_{c}\times {\text{N}}_{\text{FeedReq}\_\text{c}}$$ (6)

$${\text{N}}_{\text{Lm}1\text{stSer}}=m_{h1stSer}\times {\text{N}}_{\text{FeedReq}\_\text{h}1\text{stSer}}\times \left(1-s_{h1stSer}\right)$$ (7)

$${\text{N}}_{\text{Lm}1\text{stCal}}=m_{h1stCal}\times {\text{N}}_{\text{FeedReq}\_\text{h}1\text{stCal}}\times \left(1-s_{h1stCal}\right)$$ (8)

Finally, overall N losses from the compartment due to replacement heifer mortality were calculated with the following equation:

$${\text{N}}_{\text{Lhm}}={\text{N}}_{\text{Lmp}}+{\text{N}}_{\text{Lmn}}+{\text{N}}_{\text{Lmc}}+{\text{N}}_{\text{Lm}1\text{stSer}}+{\text{N}}_{\text{Lm}1\text{stCal}}$$ (9)

Within the heifer removal compartment, N gained from removed heifers that were sold for meat was calculated with the following equations:

$${\text{N}}_{\text{G}1\text{stSer}}={\text{BW}}_{\text{h}1\text{stSer}}\times s_{h1stSer}\times \left({\text{P}}_{\%\text{B}}/100\right)/{\text{N}}_{6.25}$$ (10)

$${\text{N}}_{\text{G}1\text{stCal}}={\text{BW}}_{\text{h}1\text{stCal}}\times s_{h1stCal}\times \left({\text{P}}_{\%\text{B}}/100\right)/{\text{N}}_{6.25}$$ (11)

$${\text{N}}_{\text{Ghm}}={\text{N}}_{\text{G}1\text{stSer}}+{\text{N}}_{\text{G}1\text{stCal}}$$ (12)

#### Pregnancy

A third compartment was used to calculate N_Lpreg_ based on N_PreReq_ for days 190 to 279 of pregnancy using equations of the 2001 Dairy NRC model [22]. Nitrogen required for pregnancy was calculated for days 190 and 279 of pregnancy and the corresponding cumulative N_PreReq_ was calculated as the area under the interpolated line. The N retained in the developing calf (N_Calf_) was calculated from BW_B_ using N_6.25_ and 16% P_%B_. Then, N_Lpreg_ was calculated with the following equation:

$${\text{N}}_{\text{Lpreg}}={\text{N}}_{\text{PreReq}}-{\text{N}}_{\text{Calf}}$$ (13)

#### Cow removal

To account for N lost through death or gained when sold for meat by removing cows from the herd (N_Lcul_ or N_Gsl_, respectively), the breed related BW_M_ was considered to be the final weight. To differentiate between cows that were removed without any use of their carcass and those that were sold for meat a constant was used (*s*_*c*_; proportion of cows sold for meat out of total animals removed). Then, N_Lcul_ and N_Gsl_ were calculated with the following equations:

$${\text{N}}_{\text{Lcul}}={\text{BW}}_{\text{M}}\times \left(1-s_{c}\right)\times \left({\text{P}}_{\%\text{B}}/100\right)/{\text{N}}_{6.25}$$ (14)

$${\text{N}}_{\text{Gsl}}={\text{BW}}_{\text{M}}\times s_{c}\times \left({\text{P}}_{\%\text{B}}/100\right)/{\text{N}}_{6.25}$$ (15)

#### Disease and fertility

Another set of equations was used to estimate opportunity costs related to health issues and were expressed in terms of a loss of MY. In the current study, opportunity costs reflect milk losses caused by diseases, disorders or sub-optimal fertility compared with full productivity from healthy, fertile cows. An early attempt to incorporate opportunity costs through milk yield reductions was described by Kossaibati and Esslemont [14]. The following issues and disorders were considered: perinatal calf mortality, retained placenta, milk fever–mild, milk fever–severe, vulval discharge, clinical mastitis–mild, clinical mastitis–severe, digital lameness, interdigital lameness and sole ulcer. Milk yield reductions for each issue and disorder are presented in Table 2 and are based on those reported by Kossaibati and Esslemont [14] and refined by Esslemont and Kossaibati [26]. The overall milk lost per cow due to disease (M_LD_) and fertility issues was calculated, based on a risk factor (r_cm_m_, r_cm_s_, r_dl_, r_il_, r_mf_m_, r_mf_s_, r_pm_, r_rp_, r_su_, and r_vd_) and milk yield losses (M_Lcm_m_, M_Lcm_s_, M_Ldl_, M_Lil_, M_Lmf_m_, M_Lmf_s_, M_Lpm_, M_Lrp_, M_Lsu_, and M_Lvd_) for each disease. For example, for retained placenta, a r_rp_ of 3.9% and estimated M_Lrp_ per cow each year of 415 kg were used (Table 2); thus, the overall opportunity loss of milk per cow and lactation was 16.2 kg (415 × 3.9 / 100). In addition, milk losses due to extended CI (M_LCI_) were calculated as a loss of 0.2% of MY daily for each day above 365 [26]. Then, M_Lop_, the sum of opportunity costs, and a specified P_%_ were used to estimate N_Lop_ at a cow level per lactation using the N_6.38_ conversion factor for milk:

$${\text{M}}_{\text{Lop}}={\text{M}}_{\text{LCI}}+{\text{M}}_{\text{LD}}$$ (16)

$${\text{N}}_{\text{Lop}}={\text{M}}_{\text{Lop}}\times \left({\text{P}}_{\%}/100\right)/{\text{N}}_{6.38}.$$ (17)

**Table 2:** Health management index used to calculate opportunity losses due to health issues (adapted from [26]).

Health problem	Milk reduction, kg / lactation^a^	Risk, cases per 100 cows^b^		
		Minimum	Maximum	Average
Perinatal calf mortality	117	5.0	9.0	8.0
Retained placenta	415	2.0	5.0	4.0
Milk fever—mild	215	1.8	9.8	7.1
Milk fever—severe	540	0.2	1.2	0.9
Vulval discharge	325	9.0	31.0	14.0
Clinical mastitis—mild	350	15.3	45.0	17.1
Clinical mastitis—severe	1050	1.7	5.0	1.9
Digital lameness	505	3.7	14.8	6.6
Interdigital lameness	160	3.4	13.7	6.1
Sole ulcer	506	1.9	7.6	3.4

^a^ Tables 4.10–4.26 in the original study [26] assuming a dairy cow with average annual milk production of 7,000 kg

^b^ Adapted from Appendix 5.1 in the original study [26], including prevalence of average milk fever (89 and 11% for mild and severe cases, respectively; Table 4.18, correcting for fatal cases that are included in mortality rates in the current study), clinical mastitis (90 and 10% for mild and severe cases, respectively; Table 4.22) and lameness (41, 38 and 21% for digital, interdigital and sole ulcer, respectively; Table 4.28)

#### Milk production

The last compartment was used to calculate N_Omilk_ from MY, P_%_ and N_6.38_. Thus, the overall N_Lmilk_ was calculated with the following equations:

$${\text{N}}_{\text{Omilk}}=\text{MY}\times \left({\text{P}}_{\%}/100\right)/{\text{N}}_{6.38}$$ (18)

$${\text{N}}_{\text{Lmilk}}={\text{N}}_{\text{Omilk}}\times \left(1/\text{MNE}–1\right)$$ (19)

where MNE represents the value for a given diet fed to the healthy cows of the herd. The major determinants of MNE is diet composition (in particular its protein concentration and fermentable energy density) and feed intake relative to productivity [9]. The former varies considerably between farms depending on feed resource availability, and for this reason we chose not to include direct feed variation in our analysis for lactating cattle, and to treat MNE as the principal input for it.

#### Herd level calculations

Once N loss of gain were calculated for each compartment, the lifetime losses were estimated taking into account the specified replacement rate (*rep*) and/or the corresponding average number of lactations for each cow in a herd (Lact = 1/*rep*). These model flows were then expressed at a herd level (where *n* is the specified number of lactating cows in the herd) and were calculated with the following equations:

$${\text{N}}_{\text{L}1\text{st}}=\left({\text{N}}_{\text{Lgr}}+{\text{N}}_{\text{Lhm}}+{\text{N}}_{\text{Lpreg}}\right)\times n$$ (20)

$${\text{N}}_{\text{Lrepl}}={\text{N}}_{\text{L}1\text{st}}+{\text{N}}_{\text{Lcul}}\times n$$ (21)

$${\text{N}}_{\text{ReplBW}}={\text{N}}_{\text{Hbody}}\times n$$ (22)

$${\text{N}}_{\text{LmilkLT}}={\text{N}}_{\text{Lmilk}}\times \text{Lact}\times n$$ (23)

$${\text{N}}_{\text{LopLT}}={\text{N}}_{\text{Lop}}\times \text{Lact}\times n$$ (24)

$${\text{N}}_{\text{LpregLT}}={\text{N}}_{\text{Lpreg}}\times \left(\text{Lact}-1\right)\times n$$ (25)

$${\text{N}}_{\text{Llact}}={\text{N}}_{\text{LmilkLT}}+{\text{N}}_{\text{LopLT}}+{\text{N}}_{\text{LpregLT}}$$ (26)

$${\text{N}}_{\text{OmilkLT}}={\text{N}}_{\text{Omilk}}\times \text{Lact}\times n$$ (27)

$${\text{N}}_{\text{OmeatLT}}=\left({\text{N}}_{\text{Gsl}}+{\text{N}}_{\text{Ghm}}\right)\times n$$ (28)

$${\text{N}}_{\text{Prod}}={\text{N}}_{\text{OmilkLT}}+{\text{N}}_{\text{OmeatLT}}$$ (29)

#### Efficiencies of N utilization

As the last step of the CNE model, N use efficiencies were calculated as follows:

$$\text{ReplNE}={\text{N}}_{\text{ReplBW}}/\left({\text{N}}_{\text{ReplBW}}+{\text{N}}_{\text{Lrepl}}\right)$$ (30)

$$\text{LactNE}={\text{N}}_{\text{OmilkLT}}/\left({\text{N}}_{\text{OmilkLT}}+{\text{N}}_{\text{Llact}}\right)$$ (31)

$$\text{LNE}={\text{N}}_{\text{Prod}}/\left({\text{N}}_{\text{Prod}}+{\text{N}}_{\text{Lrepl}}+{\text{N}}_{\text{Llact}}\right)$$ (32)

### Model sensitivity analysis

The sensitivity of the model to variation in farm- and animal-related input values was evaluated with a Monte Carlo simulation using @Risk version 7.1 (Palisade, West Drayton, UK). Values for a modelled dairy farm were used based on published data related to dairy farming practices in the United Kingdom [27–33]. To assist reporting net values of main N outputs, an example herd of a fixed size of 100 lactating dairy cows, plus the heifers needed to replace these cows, was modelled. Male and surplus female calves were assumed to be sold at birth to be reared elsewhere. Productive N output in milk and cull-cow and heifer meat (in 1000s of kg) was calculated for the whole herd over the average animal’s lifetime and each output was expressed as a percentage of the total. Probability density functions were fitted to farm and animal input values. Table 3 describes tested variables, their type of distribution and their selected values. All variables and their range were evaluated for their biological correctness and correlation. For example, the onset of puberty is determined by BW as heifers start to cycle at approximately 43% of mature BW[33]. We used a mature BW of 748 kg that requires BW at first service of about 321 kg. In our dataset the minimum BW at first service is 320 kg. Moreover, we chose to use a MNE value (0.277) reported by Huhtanen and Hristov [9] for the North European dataset that reflected diets similar to those used in the UK within a similar MNE range [34–37].

**Table 3:** Distribution characteristics of inputs used in Monte Carlo sensitivity analysis.

Variable	Normal		Non-normal^a^			Ref
	Mean	SD	Max	Likely	Min	
Annual milk yield (MY), kg			7870	7096	6449	AHDB^b^
BW^c^ at first calving (BW_1stCal_), kg	544	25				[33]
BW at first service (BW_1stSer_), kg	368	29				[30]
BW mature (BW_M_), kg	748	75				[18]
BW at birth (BW_B_), kg	43.4	4.9				[33]
Calf mortality rate (m_c_)	0.034	0.036				[29]
Calving interval (CI), d			600	385	365	[27]
Cattle replacement rate (*rep*)			0.287	0.238	0.175	[31, 32]
Heifer mortality rate to first calving (m_h1stCal_)	0.037	0.05				[29]
Heifer mortality rate to first service (m_h1stSer_)	0.032	0.046				[29]
Milk nitrogen use efficiency (MNE)	0.277	0.036				[9]
Milk protein content (P_%_),%	3.21	0.17				[9]
Neonatal mortality rate (m_n_)	0.032	0.040				[29]
Perinatal mortality rate (m_p_)	0.081	0.036				[29]
Sold rate of dairy cattle (s_c_)	0.93	0.01				[32]
Sold rate of heifers to first calving (s_h1stCal_)	0.95	0.09				[29]
Sold rate of heifers to first service (s_h1stSer_)	0.19	0.02				[29]
Age at weaning (T_w_), d	42	4.2				[29]
Age to first calving (T_1stCal_), m			50.9	26.4	21.2	[28]
Age to first service (T_1stSer_), d			963	473	357	[28]

^a^ The triangular distribution was used for age to first calving and age to first service, and the program evaluation and review technique (PERT) for annual milk yield, calving interval, and cattle replacement rate.

^b^ From Agriculture and Horticulture Development Board (AHDB) using average annual production. Then, SD reflects annual variation and not cow-herd variation; http://dairy.ahdb.org.uk/market-information/farming-data/milk-yield/average-milk-yield

^c^ BW: body weight

**Fig 2:**
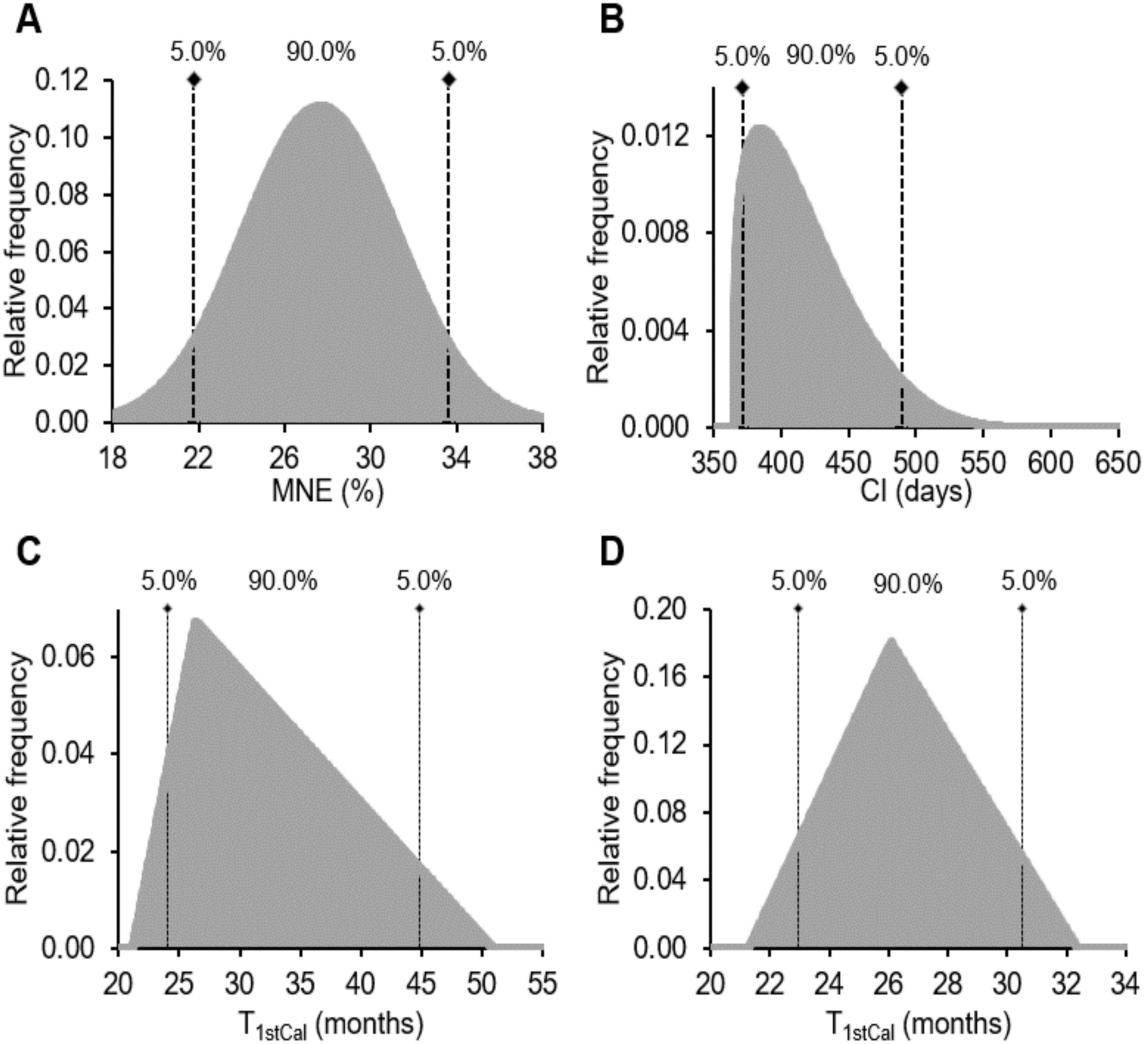
Frequency distributions of major inputs used in Monte Carlo sensitivity analysis. (A) Normal distribution of milk nitrogen use efficiency (MNE), (B) program evaluation and review technique (PERT) distribution of calving interval (CI), (C) triangular distribution of time to first calving (T_1stCal_) based on Brickell et al. [28], (D) triangular distribution of T_1stCal_, based on Boulton et al.[40].

Most variables were described with a normal distribution except those for which limited or apparently extreme data were available (e.g. annual milk production, where the Agriculture and Horticulture Development Board (AHDB) annual data were used, representing the country’s annual variation rather than cow-herd variation) or when the variable is not distributed normally (e.g. T_1stCal_ [38]). In this case, we used either the triangular or the Program Evaluation and Review Technique (PERT) distribution. Both distributions require 3 estimates: (i) the most likely result, (ii) the minimum expected result, and (iii) the maximum expected result. With the triangular distribution values around the most likely result are more likely to occur. The PERT distribution is similar to a β or triangular distribution and is useful to describe variation in a situation where limited data exists [39]. The distribution of selected inputs is presented in Fig 2. We used contemporary peer-reviewed data to build our dataset where the range of T_1stCal_ was up to 50 months [28]. However, a recently published study analysing the cost of heifer growth in the UK reported a narrower range than the one we used in our analysis, where T_1stCal_ ranged from 21.3 to 32.4 with a mean of 26.1 months [40]. Therefore, we performed the same sensitivity analysis but using the values of the later study for T_1stSer_ (509, 365, and 700 days of age for most likely, minimum expected and maximum expected result, respectively) and T_1stCal_ [40]. In both cases, triangular distributions were considered in our analysis for T_1stSer_ and T_1stCal_ as suggested by published data [38].

In addition, dietary inputs for growing animals necessary to run the simulation were obtained using the CNCPS assuming animals were grazing perennial ryegrass with varying CP concentrations. Three CP concentrations of ryegrass were considered (10, 16 and 20% on a dry matter basis) and the chemical composition necessary to run a simulation with the CNCPS was obtained from the literature [36, 37, 41–44] and the CNCPS feed library. Simulations were run for each CP concentration and animal type (heifers at first service and heifers at first calving) using animal inputs (body weight and age) reported in Table 3 for each stage, and feed dry matter intake as predicted by the CNCPS (9.4 and 12.6 kg of dry matter intake daily for first service and first calving heifers, respectively). Then metabolizable protein supply was calculated by the CNCPS and the ratio of metabolizable protein supply to crude protein intake (MP:CP) was estimated. Due to feeding similarities for both stages, MP:CP_1stSer_ and MP:CP_1stCal_ were similar. Therefore, a merged factor was used (MP:CP_heifer_) and variation in MP:CP_heifer_ was described with a PERT distribution using the following values reflecting feed variation: (i) minimum expected result = 0.505, obtained by feeding ryegrass with a CP concentration of 20% on a dry matter basis, (ii) most likely result = 0.605, obtained by feeding ryegrass with a CP concentration of 16% on a dry matter basis, and (iii) maximum expected result = 0.850, obtained by feeding ryegrass with a CP concentration of 10% on a dry matter basis. Frequency distributions for model outputs were generated using a Monte Carlo simulation with 10,000 iterations to describe the range of possible outcomes for each output and the relative likelihood of occurrence.

## Results

**Fig 3:**
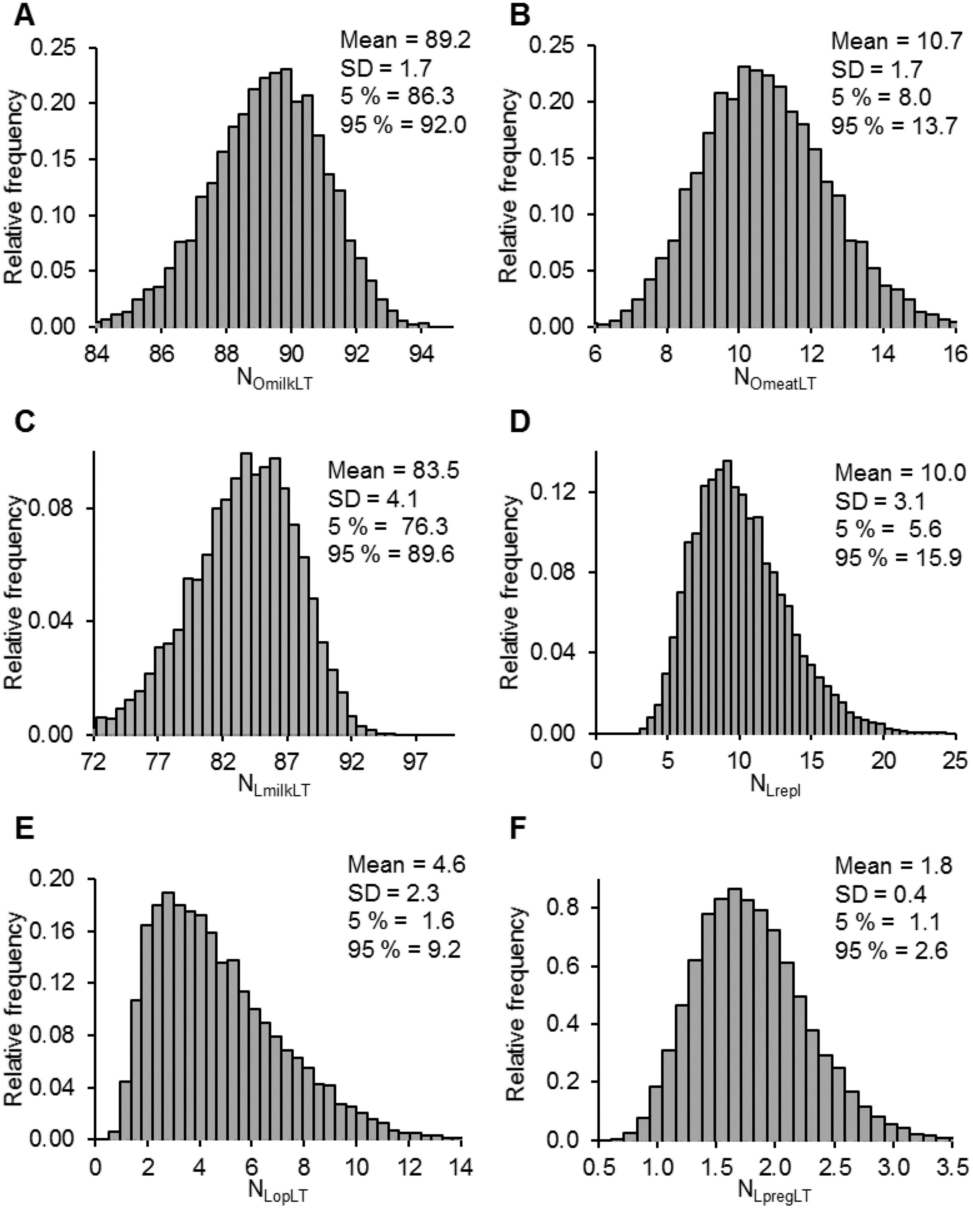
Frequency distributions of major lifetime nitrogen outputs and losses at a herd level for lifetime expressed as % of total N losses in lifetime. (A) Nitrogen output in milk (N_OmilkLT_), (B) Nitrogen output in meat (N_OmeatLT_), (C) Nitrogen lost for milk production (N_LmilkLT_), (D) Nitrogen lost for replacement cattle (N_Lrepl_), (E) Opportunity nitrogen losses (N_LopLT_), and (F) Nitrogen lost for pregnancy (N_LpregLT_).

Productive N outputs over the course of an animal’s lifetime were partitioned into milk (N_OmilkLT_) and meat (N_OmeatLT_), and they were dominated by milk production. Indeed, N_OmilkLT_ represented on 89% of total N output, and the remainder 11% was partitioned in N_OmeatLT_ (Fig 3). As presented in Table 4 for the modelled farm of 100 lactating dairy cows, a net production between 12,700 and 18,400 kg of N_OmilkLT_ was estimated, with the range being most significantly affected by variation in cattle replacement rate, milk protein concentration and milk yield. Similarly, a total production between 1,420 and 2.280 kg of N_OmeatLT_ was calculated, with the range being most significantly affected by variation in cow sold rate, mature body weight, and heifer mortality to first calving.

**Table 4:** Factors affecting nitrogen (N) gains and losses (values in 1000s of kg in lifetime at a herd level), corresponding effects on output means, and their correlation coefficients (r).

Item^a^^,^^b^	Mean	SD	5%	95%	Effect on output mean		*r*
					Form	To	
N_OmilkLT_	15.4	1.7	12.7	18.4			
* rep*					13.2	18.0	-0.81
P_%_					13.9	16.9	0.45
MY					14.3	16.3	0.32
N_OmeatLT_	1.83	0.26	1.42	2.28			
* s*_*c*_					1.51	2.14	0.67
BW_M_					1.52	2.14	0.66
m_h1stCal_					1.72	1.93	0.22
N_LmilkLT_	41.0	9.0	28.6	57.6			
MNE					29.8	56.7	-0.84
* rep*					35.3	47.8	-0.42
P_%_					37.3	45.0	0.23
MY					38.2	43.5	0.17
N_Lrepl_,	4.76	1.35	2.86	7.25			
T_1stCal_					3.08	6.96	0.88
MP:CP_heifer_					3.91	5.53	-0.36
m_h1stCal_					4.34	5.21	0.19
* s*_*c*_					4.43	5.03	-0.14
m_h1stSer_					4.55	4.95	0.10
N_LopLT_	2.24	1.17	0.378	4.50			
CI					0.81	4.61	0.97
* Rep*					1.92	2.59	-0.18
Health index					2.06	2.59	0.12
N_LpregLT_	0.85	0.20	0.53	1.17			
* rep*					0.61	1.07	-0.66
BW_M_					0.65	1.05	0.58
BW_B_					0.79	0.91	-0.17

^a^ N_OmilkLT_: nitrogen output in milk for lifetime, N_OmeatLT_: nitrogen output in meat for lifetime, N_LmilkLT_: nitrogen lost for milk in lifetime, N_Lrepl_: nitrogen lost for replacement cattle, N_LopLT_: opportunity nitrogen losses, N_LpregLT_: nitrogen lost for pregnancy in lifetime, *rep*: cattle replacement rate, P_%_: milk protein content, MY: annual milk yield, *s*_*c*_: sold rate of dairy cows, BW_M_: mature body weight, m_h1stCal_: heifer mortality rate from first service to first calving, MNE: milk nitrogen use efficiency, T_1stCal_: age at first calving, MP:CP_heifer_: metabolizable protein to crude protein ratio for heifer diet, m_h1stSer_: heifer mortality rate from six months to first service, CI: calving interval, BW_B_: body weight at birth

^b^ Factors that affect a variable were listed when r ≥ ± 0.1

However, this overall production was achieved with substantial N losses. Nitrogen lost during lifetime milk production at a herd level were on average 41,000 kg but may reach 57,600 kg for a 100-cow dairy (Table 4), mainly affected by milk N use efficiency, replacement rate and production characteristics, such as milk protein concentration and milk yield. Nitrogen losses incurred by replacing dairy cows within the herd represented a lower portion of losses (mean = 4,760 ± 1,350 kg of N_Lrepl_) than those during lifetime milk production and were strongly affected by the last time point of heifer growth (T_1stCal_ and its related mortality rate) rather than feed variation, as assessed by variation in MP:CP_heifer_. Even though the overall contribution of N_LopLT_ was relatively small compared with N losses during lactation, it was estimated to be between 530 and 1,170 kg, mainly being affected positively by variation in calving interval and disease index, and negatively by variation in cattle replacement rate. Further, a much lower proportion of N losses were partitioned in pregnancy (mean = 850 kg of N_LpregLT_), and these were positively affected by variation in mature body weight and negatively by variation in replacement rate and body weight at birth.

**Fig 4:**
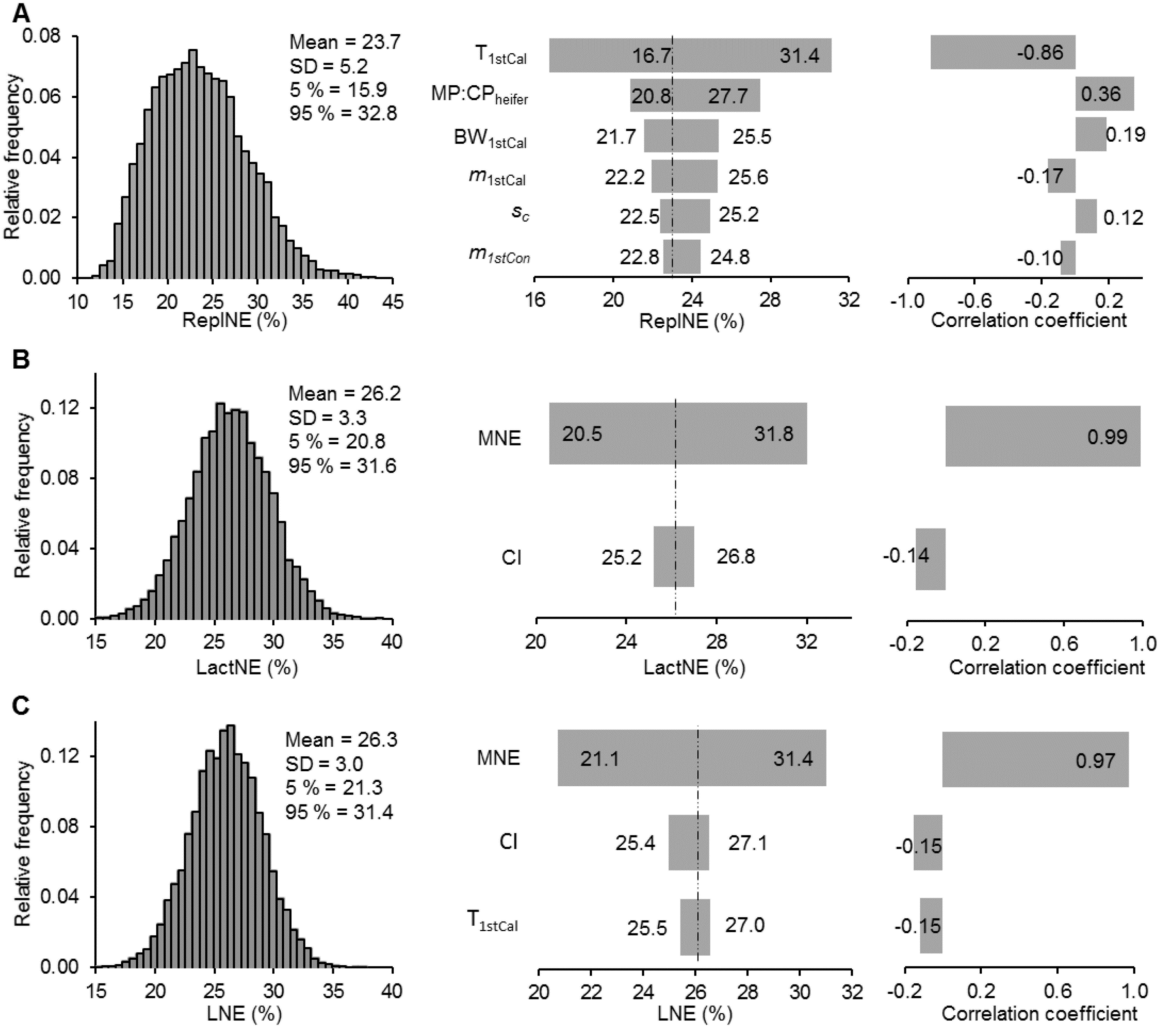
Frequency distributions and tornado diagrams showing the change in means outputs and correlation coefficients of overall lifetime use efficiencies. (A) Replacement nitrogen use efficiency (ReplNE). (B) Lactation nitrogen use efficiency (LactNE). (C) Lifetime nitrogen use efficiency (LNE). Where, MNE: milk nitrogen use efficiency, CI: calving interval, T_1stCal_: age at first calving, MP:CP_heifer_: metabolizable protein to crude protein ratio of feed fed to heifers after 100 kg of body weight, *m*_1stCal_: heifer mortality rate from first service to first calving, BW_1stCal_: body weight at first calving, *s*_c_: sold rate for dairy cows, *m*_1stSer_: heifer mortality rate from six months to first service.

Replacement heifers form an important part of the dairy herd in terms of animal numbers and overall cost. With the CNE model we estimated a mean ReplNE of 23.7%, which was most substantially affected by the last stage of heifer growth. Variation in time to first calving may cause larger changes on ReplNE than variation in feed as assessed by the MC:CP_heifer_ (Fig 4A). Both weight and heifer mortality to first calving were correlated with ReplNE: positively with BW_1stCal_ (*r* = 0.19) and negatively with m_1stCal_ (*r* = -0.17). The sensitivity analysis showed a very strong positive correlation between LactNE and MNE (*r* = 0.99; Fig 4B). Variation in MNE was found to cause changes in LactNE mean from 20.5 to 31.8%. However, a small negative relationship of opportunity losses was found with extended calving interval (*r* = -0.14). Within the structure of the CNE model, the combination of the efficiency of N use by replacement animals (i.e. ReplNE) and the efficiency of use of N for lactation (i.e. LactNE) is expressed in LNE. We estimated a mean of 26.3% for LNE (Fig 4C), and it was dominated by MNE as indicated by *r* = 0.97. However, our study highlighted two other model variables that affected LNE. Variation in CI (*r* = −0.15) and T_1stCal_ (*r* = −0.15) may cause measurable reductions of overall LNE. A recent study [40] published T_1stCal_ values that were less variable than those of previous studies used to define the frequency distributions for initial Monte Carlo simulations. Therefore, we performed an additional sensitivity analysis using these new values. In this case, reduced variation in T_1stCal_ indicated weaker effects in mean LNE provoking mean changes from 26.5 to 27.1% (*r* = −0.04), but remained the principal variable responsible for changes in mean ReplNE (from 24.3 to 32.5%; *r* = −0.59). In addition, the mean values of both ReplNE and LNE increased to 28.1 ± 3.9% and 26.8 ± 3.1%, respectively.

## Discussion

Despite the European dairy herd’s main role as a producer of milk, is also a very important source of beef meat, with approximately 50% of produced beef estimated to come from culled dairy cows and 15% from male dairy calf systems [45]. In 2014, the EU-28 countries produced 151.7 million tonnes of liquid bovine milk with an average protein concentration of 3.37%, resulting into 0.805 million tonnes of milk N being produced [46]. For the same period, the overall bovine carcass production was 7.59 million tonnes and assuming an average carcass protein concentration of 16.5%, the overall N output in bovine meat production was 0.200 million tonnes [46]. Considering approximately 50% of this was from culled dairy cows [45], we can estimate meat N output at 11% of total N output from dairy cattle. This matches very well the calculations in the current study of meat N output being 10.7% of total N produced by a dairy farm, for an annual MY range from 6,449 to 7,870 kg/cow (Table 3).

With this modelling exercise and by describing the UK dairy sector, we estimated significant lifetime N losses at a herd level of up to 57,600 kg of N for a 100-cow dairy herd. This may happen in a high-yielding herd with high milk protein content, low replacement rate but which produce milk protein using dietary nitrogen with a low rate of efficiency. For example, a 100-cow herd with 7000 kg /cow milk produced per year with 3.5% milk protein content, a replacement rate of 0.25 (4 lactations) and a milk nitrogen use efficiency of 0.22, will excrete into the environment 54,460 kg of N in lifetime. If we calculate the daily N excretion of the lactating cow for this herd (total excretion / lactations / *n* / 305 days of milk production) we will estimate a daily N excretion of 446 g / cow, which is within the normal range of N excretion reported in the literature [9, 18, 34]. Moreover, it should be noted that in the current analysis replacement rate is negatively correlated with lifetime N excretion. However, this is mainly related to the lifetime calculation of excretion and does not indicate a recommendation for higher replacement rate.

The majority of lifetime N losses were accounted for by losses in the milk production process. Besides the importance of milk production of the dairy herd in terms of total outputs, this is also because the efficiency of converting feed N into milk N is relatively poor, with a large proportion of feed N being excreted in faeces and urine (on average 72% of N intake) [11]. Variation in MNE was the principal cause of changes in N_LmilkLT_ and this was reflected in LactNE and consequently in overall LNE, which was highly correlated with MNE. In the current study, we considered baseline MNE to be the efficiency of N utilization for milk protein production for healthy lactating animals within the herd. Several studies have reported MNE, ranging from 14.0 to 45.3% [9–11] and reaching a theoretical maximum of about 45% for a 600 kg dairy cow producing daily 25 kg of milk [47]. The major determinant of MNE is nutrition [9], and in our sensitivity analysis reflects different feeding scenarios and production levels in the UK. Use of the model at a farm level will require knowledge of that farm’s baseline MNE as an input. However, the model could be linked to nutritional models (e.g. CNCPS) that could provide MNE predictions based on different feeding scenarios.

Besides the dominant effects of MNE on LNE, we detected the effects that variation in specific non-diet related variables have on changes in LactNE and LNE means. In particular, variation in CI may cause measurable changes in LactNE and LNE. To our knowledge, this study is the first to have assessed the implications of factors such as CI with the effects of opportunity costs of disease and infertility leading to N losses during lactation. Traditionally, CI has been used as an indicator of herd fertility [48, 49] and a short CI of 10 to 12 months (300–365 days) has been recommended for maximizing herd profitability [50, 51]. However, several studies have questioned this approach, suggesting that a longer CI, even of up to 24 months, may be beneficial either as a practice to avoid high replacement rates caused by infertility in seasonal-calving-based systems [52, 53], or for high-producing dairy cows that are dried off with more than 25 kg/d of milk production [54].

An extended lactation length, and therefore an extension of CI, may increase yields per lactation but will depress annualized MY (expressed on a 305-day basis) by delaying the following lactation [26]. This demonstrates the opportunity costs due to extended CI in the current study. We calculated annual MY (305-day) reductions for each extra day of CI above 365 days, based on the best alternative which is calving in 365-day cycle, considering a 12 months CI to be the standard management decision within our dataset [27]. Therefore, milk opportunity cost reflects the theoretical additional amount of milk that would have been produced if cows had been in a following lactation assuming a typical lactation curve, which increases rapidly from calving to a peak at about 6 weeks of lactation and then decreases gradually as lactation progresses [55].

Reduced annualized MY (= MY × 12 / CI) up to 10.5% for cows with 24 months CI compared with those having a CI of 12 months was reported [53]. In a following study, cows with extended CI (24 months) produced 7.1% less milk in two years compared with the 12 months CI group [56]; in terms of annualized MY, cows with extended CI produced 22% less milk. Similarly, pasture-based cows with 24 months CI produced on average 21% less milk during the second year (13 to 24 m) compared with the first year (1 to 12 m) [52], suggesting an opportunity cost of 21% for the extended CI. These findings are in accordance with our results, where opportunity costs of milk production due to CI averaged 10.5% of annual milk yield (results not shown) for extended CI from 366 to 600 days (Table 3). Thus, these losses resulted in a negative correlation with both LactNE and LNE.

A negative correlation with both LNE and ReplNE was found for T_1stCal_, which is an indicator of replacement cattle growth and fertility efficiency. This suggested that the efficiency by which replacement heifers are grown affects overall LNE. Replacement heifers represent a major economic expense for dairy operations, being the second largest input after feed costs, and accounting for 15 to 20% of total milk production costs [57]. Several studies in the USA and Europe suggested that T_1stCal_ is the primary variable to define net cost for replacement cattle [58, 59]. Within the structure of our model, T_1stCal_ affected total feed N requirements for heifer growth (in the model: N_FeedReq_) and consequently N losses for growth for the period between first service and first calving. A mean N_FeedReq_ of 42.9 ± 5.6 kg of N per heifer for a BW_1stCal_ of 544 kg (Table 3) was estimated, suggesting a feed N utilization efficiency of 25.2%, which is within the range reported in the literature [17]. Feed N requirements for the heifer between first service and first calving was 44% of N_FeedReq_. Similarly, for the period between BW_100_ and first service, feed N requirements were 49% of N_FeedReq_, but variation in T_1stSer_ did not cause significant changes in either LNE or ReplNE. This is because we calculated N_FeedReq_ as a cumulative growth function of MP requirements in time for three stages of heifer growth after weaning (BW_100_, first service and first calving). Using this approach, MP requirements for a heifer at first calving is 28% higher than those for a heifer at first service due to BW differences. For this reason, variation in T_1stCal_ led to larger changes in N_FeedReq_ (from 36.1 to 50.3 kg of N per heifer) than variation in T_1stSer_ (from 42.0 to 43.8 kg of N per heifer), and consequently to significant changes in ReplNE (Fig 4).

This variation in T_1stCal_ does not reflect growth variation only but incorporates variation in fertility of the replacement heifers as well. Once heifers have reached an adequate BW_1stSer_ they are ready for breeding, but they rarely conceive immediately. A mean of 3 services before conception, with a range between 1 and 9, was reported for the UK [29]. The delay in conception increases the time to first calving, extending the time between T_1stSer_ to T_1stCal_ beyond 280 days. Therefore, an overall improvement of T_1stCal_ can be achieved from both better heifer growth rates and improved conception rates. Interestingly, variation in T_1stCal_ caused larger changes in ReplNE than feed variation as included by MP:CP_heifer_. In our analysis we avoided the inclusion of a wide variety of feeding strategies for heifers and we focused on grazing systems as the principal strategy in the UK. Links of the CNE model to feed models, such as the CNCPS, could be used to incorporate and analyse this variation in future work. Variation in heifer mortality was negatively correlated to ReplNE. Variation in mortality rates from first service to first calving caused larger changes to ReplNE than variation in mortality rates from weaning to first service, probably because the first time period includes heavier heifers leading to higher N losses due to mortality.

In any sensitivity analysis, the results depend on the range of values used as inputs. Different studies describing the UK dairy sector have reported different range for T_1stSer_ and T_1stCal_ [28, 40] We used contemporary peer-reviewed data to build our dataset with a range of T_1stCal_ up to 50 months [28]. This may be considered extreme for modern dairy farming, even though this high value may include extensive dairy systems that do exist in the UK. Even though the most likely value, according to both studies, was similar (approximately 26 months for T_1stCal_) the maximum likely value was very different. Boulton et al. [40] reported a maximum T_1stCal_ of 32.4 months, which is similar to that reported for Holstein heifers in the USA in 2004 [38]. We cannot confirm if this narrower range from earlier data reflects an improvement of UK dairy farming practices or the description of different population sample. In any case, these findings suggest that better management strategies that reduce the range of T_1stCal_ may help reduce N losses during heifer growth due to extended T_1stCal_. If all heifers calved for the first time by about 32 months, LNE would be practically insensitive to replacement heifer variables.

The aim of this work was to describe whole-lifetime N use efficiency, which, by definition, limits the applicability of the model. For example, cattle replacement rate was found to be negatively correlated with lifetime N excretion but had no effect on LNE; a higher replacement rate reduces lifetime N excretion because fewer lactations are included. However, there was no effect on LNE because it is a ratio, and both numerator and denominator variables are assessed for the same number of lactations. Similarly, herd size was considered to be unchanged in the current study, and even though herd size is a dynamic variable [60, 61] that might be beneficially included for production and policy matters, its effect remains to be incorporated in future work.

## Conclusion

With the current study, we developed a dairy cattle herd model that is sensitive to elements of performance, fertility and health. Lifetime N use efficiency of dairy cattle was shown to be dominated by MNE, the short-term efficiency of use of feed N for milk production. However, we have demonstrated important effects of both the replacement cattle growth period and the opportunity costs of disease and fertility on N use efficiency. The considerable economic cost of the replacement cattle part of the dairy herd is well established. Here we demonstrated that replacement cattle have a considerable impact in terms of farm N losses. Further, we detected specific non-diet related variables that affect the efficiency of use of N in the growth of replacement cattle (ReplNE) and during lactation (LactNE), and therefore overall lifetime N use efficiency (LNE) of dairy cattle.
